# Ultrathin Endoscope-Assisted Wire-Guided Dilation for Radiation-Induced Esophageal Obstruction: A Case Report

**DOI:** 10.7759/cureus.110444

**Published:** 2026-06-08

**Authors:** Mahinaz Mohsen, Hamza R Khan, Rohan Karkra, Jasmine Baste, Weizheng Wang

**Affiliations:** 1 Internal Medicine, Rutgers University New Jersey Medical School, Newark, USA; 2 Medicine, Rutgers University New Jersey Medical School, Newark, USA; 3 Gastroenterology and Hepatology, Rutgers University New Jersey Medical School, Newark, USA

**Keywords:** endoscopic dilation therapy, esophageal stenosis, oesophageal recanalization, radiation-induced stricture, ultrathin endoscope, wire-guided dilation

## Abstract

We present a case of near-complete upper esophageal obstruction secondary to radiation necrosis in a patient with a history of hypopharyngeal squamous cell carcinoma treated with radiation and cisplatin/docetaxel chemotherapy. Standard endoscopic passage was not possible due to the absence of a visible lumen. An ERCP (endoscopic retrograde cholangiopancreatography) guidewire was advanced across the stenosis with moderate resistance, and using the wire as a guide, an ultrathin endoscope (4.9 mm; Olympus Corporation, Tokyo, Japan) was used as a dilator, achieving successful dilation to 10 mm and enabling passage into the middle third of the esophagus. Following the procedure, the patient tolerated a liquid diet and gained 13 pounds. Unlike prior reports in which the ultrathin endoscope served as an access tool followed by separate mechanical dilation or combined antegrade-retrograde dilation (CARD), in this case, the ultrathin endoscope itself functioned as the dilator, simplifying the procedure. This case highlights a resource-efficient approach to managing esophageal obstruction with an ultrathin endoscope as the dilator, offering a practical alternative in settings without access to fluoroscopy, stenting, or advanced interventional equipment. This technique may be most applicable to early inflammatory strictures rather than mature fibrostenotic ones. Findings are preliminary and based on a single case; further prospective studies are needed to establish safety, efficacy, and long-term outcomes.

## Introduction

Dysphagia and esophageal obstruction are frequent complications following treatment for head and neck cancers. Although relatively understudied, available data suggest that approximately 5% of patients undergoing head and neck radiation therapy will develop complete esophageal obstruction (CEO), while esophageal strictures occur in 3-4% of such patients [1,2]. A CEO is typically defined as an obstruction with no visible lumen and an inability to pass a standard endoscope, or even a guidewire, through the obstruction [3,4]. Since, in our case, the standard and ultrathin endoscopes did not pass, but a guidewire was inserted with moderate resistance, we define this presentation as near-complete esophageal obstruction.

The standard diagnostic workup for esophageal obstruction typically includes upper endoscopy for direct visualization and tissue sampling, barium esophagram to characterize stricture length and morphology, and cross-sectional imaging to evaluate extrinsic involvement [5]. Radiation-induced esophageal stenosis poses significant management challenges due to the lesion's location, distorted anatomy, and the friable nature of irradiated tissue. Several established techniques exist for managing esophageal obstruction depending on etiology and severity. For benign esophageal strictures, the standard of care is endoscopic dilation using bougie or balloon techniques [5]. Malignant obstruction is typically managed with self-expanding metal stents (SEMS). However, in cases of complete or near-complete obstruction, these standard dilation techniques are often ineffective, as the absence of a visible lumen prevents safe passage of a guidewire or dilator without first establishing access. For CEO, the preferred technique is therefore combined antegrade-retrograde endoscopic recanalization and dilation (CARD) [6,7]. CARD requires the simultaneous use of two endoscopes; one passed antegrade through the mouth and one retrograde through a gastrostomy tract, and is restricted to centers with a high level of expertise, given its technical complexity [6]. Additionally, patients are typically placed under general anesthesia, and the procedure requires fluoroscopic guidance to coordinate the two endoscopic approaches [7], making it resource-intensive and less accessible in community or low-resource settings.

We present a case of a man with hypopharyngeal squamous cell carcinoma who developed dysphagia after chemotherapy and radiation therapy and was subsequently found to have near-complete esophageal obstruction that was successfully managed using only a guidewire and an ultrathin endoscope. While prior reports describe the use of ultrathin endoscopy for esophageal obstruction, these cases use the ultrathin endoscope as an access tool, followed by separate mechanical dilation [8,9]. In our case, the ultrathin endoscope itself served as the dilator, with no additional dilation devices required. This modified approach offers a practical alternative for managing near-complete esophageal obstruction while avoiding the technical complexity and resource demands of CARD.

## Case presentation

We present the case of a 66-year-old male with a history of hypopharyngeal cancer treated with radiation and cisplatin/docetaxel chemotherapy (completed three months before presentation), complicated by radiation necrosis with recent tracheostomy creation and PEG (percutaneous endoscopic gastrostomy) tube placement secondary to airway swelling and poor oral intake, who presented with three weeks of progressively worsening dyspnea and intermittent cough with blood-streaked sputum. The patient was also found to have persistent dysphagia and odynophagia, making it difficult for him to tolerate his secretions, which exacerbated his cough. He was afebrile and hemodynamically stable.

**Figure 1:**
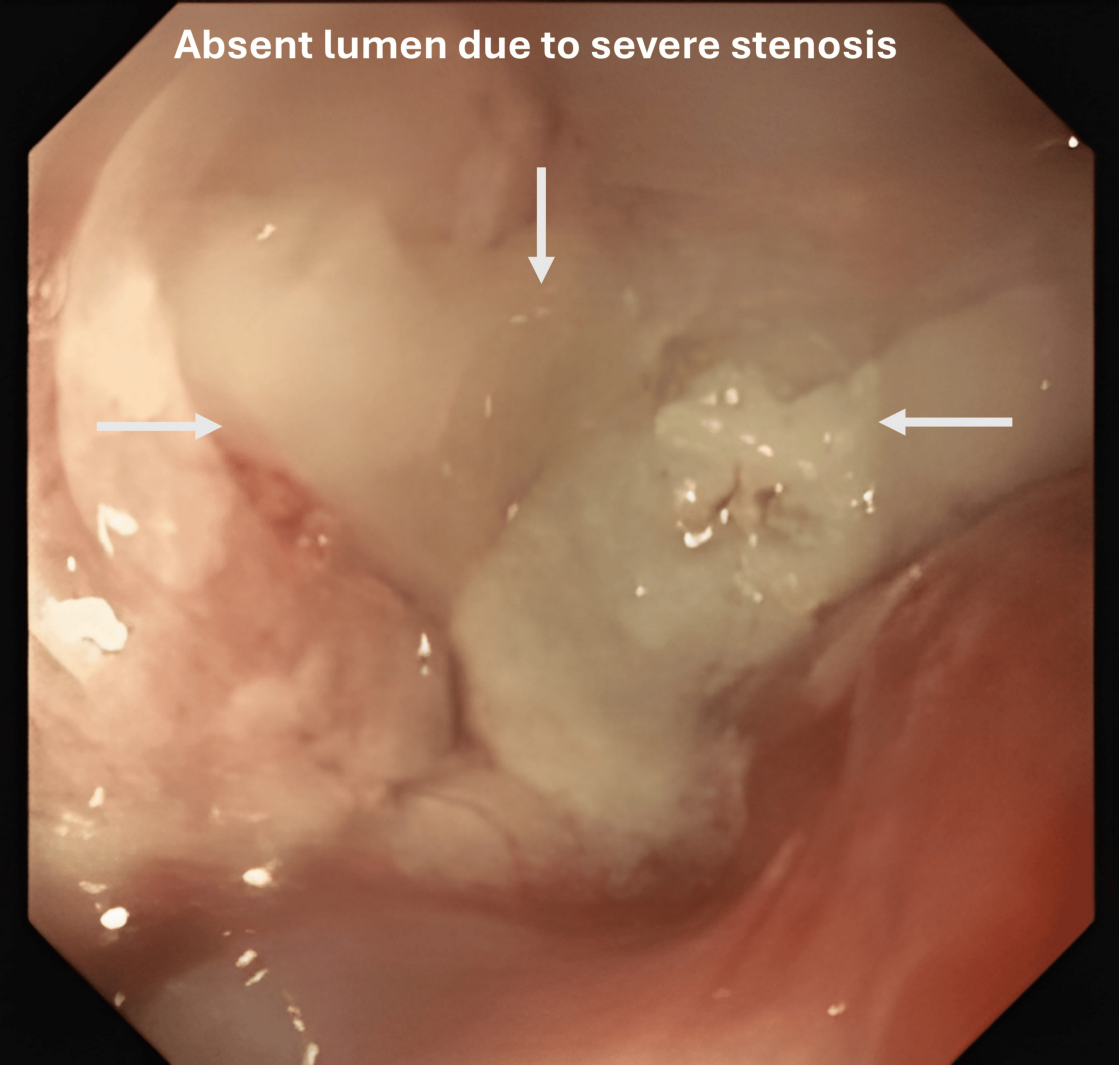
Near-Complete Upper Esophageal Obstruction Endoscopic view demonstrating near-complete esophageal obstruction with circumferential edematous mucosa and thick exudate prior to intervention. Arrows indicate the area of absent visible lumen.

Labs are notable for a hemoglobin of 6.9 g/dL. A CT chest is negative for acute pulmonary embolism or pneumonia. ENT performed a flexible laryngoscopy/tracheoscopy, which did not reveal any airway obstruction or bleeding. A modified barium swallow study demonstrated severely impaired swallowing mechanics, including absent epiglottic and hyoid movement, poor pharyngeal squeeze, decreased laryngeal closure with penetration on all swallowing attempts, and aspiration with thin liquids. These findings were consistent with severe radiation-induced dysphagia and confirmed the patient's inability to safely tolerate oral intake. GI was consulted for dysphagia and acute-on-chronic anemia, with plans for esophagogastroduodenoscopy (EGD) with PEG reinsertion, as the initial stoma was closed. EGD done under minimal sedation using propofol revealed intrinsic severe stenosis in the upper third of the esophagus, which was too narrow for a regular endoscope to pass, as no lumen was visualized (Figure 1).

**Figure 2:**
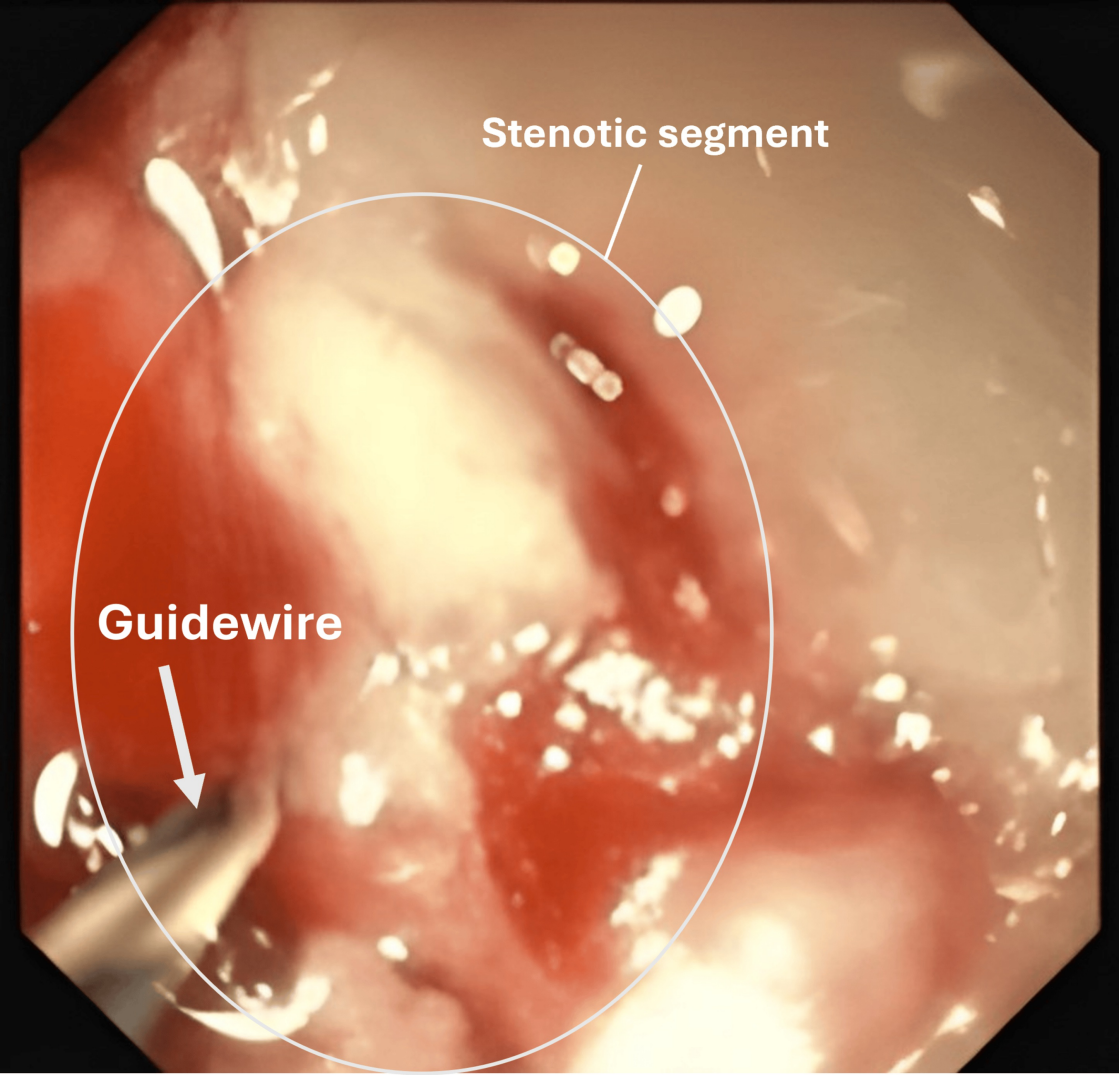
ERCP Guidewire Traversing the Stenosis Endoscopic view demonstrating ERCP guidewire passage across the stenotic segment. The circle delineates the stenotic segment, and the arrow indicates the guidewire used to direct the ultrathin endoscope, which served as the dilator.

Given the absence of a visible lumen and the inability to pass a standard endoscope, an ultrathin endoscope (4.9 mm; Olympus Corporation, Tokyo, Japan) was selected; however, traversing the stenosis remained challenging because of the thick exudate. An O25 ERCP (endoscopic retrograde cholangiopancreatography) guidewire was then selected, given its atraumatic, hydrophilic tip, which is specifically designed to navigate tight strictures while minimizing the risk of false lumen creation or perforation. The guidewire was advanced across the stenosis with moderate resistance using controlled movements, with attention to tactile feedback to differentiate the expected resistance of the stenosis from a sudden loss of resistance that would suggest perforation or a false passage (Figure 2).

**Figure 3:**
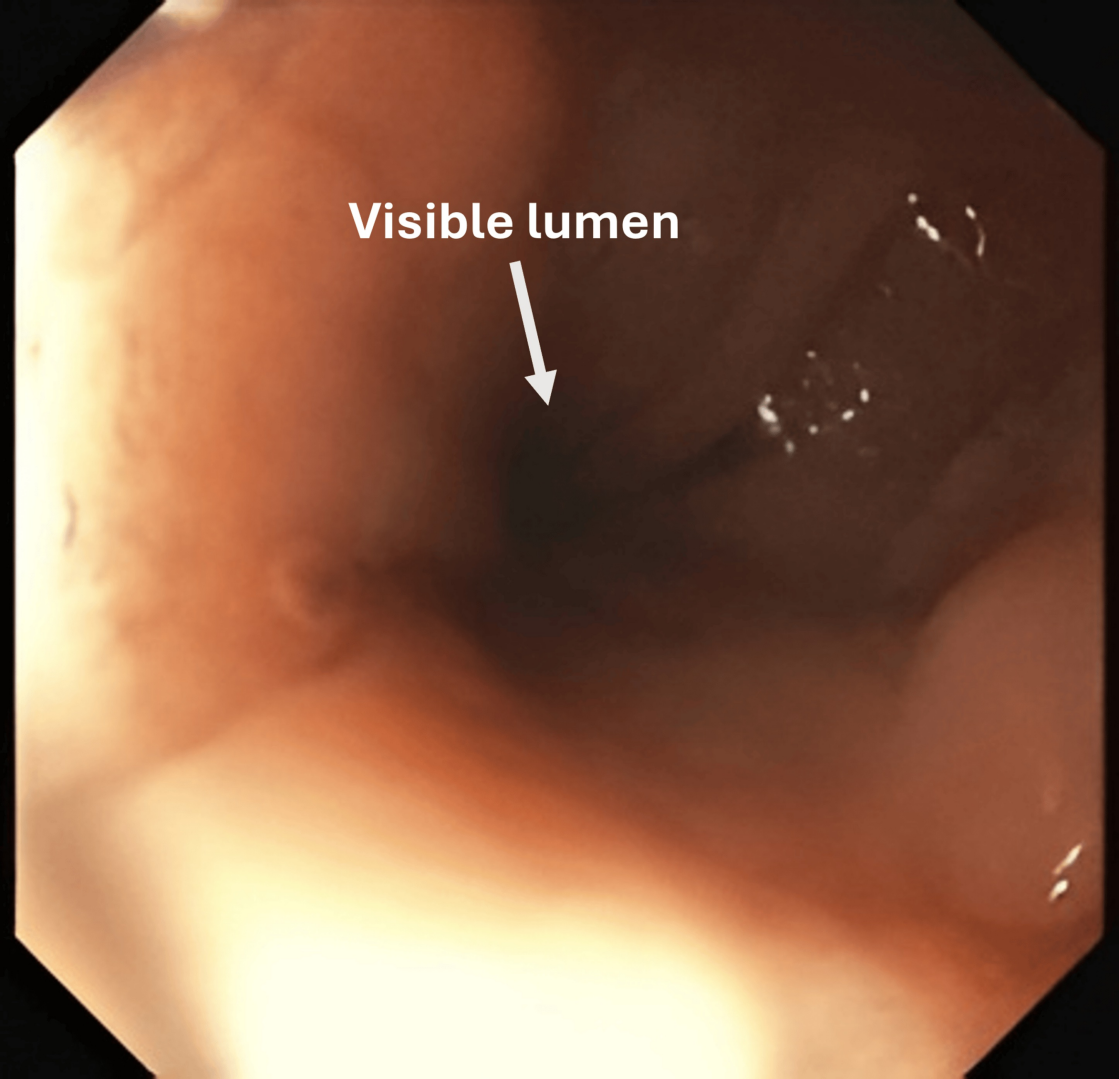
Restored Esophageal Patency Post-Dilation Post-dilation endoscopic view showing successful restoration of esophageal patency following ultrathin endoscope-guided dilation to 10 mm. The arrow indicates the visible lumen, with visualization extending into the middle third of the esophagus.

Fluoroscopic guidance was not available during the procedure. Therefore, correct guidewire placement was verified functionally: the ultrathin endoscope was advanced over the wire and successfully traversed the obstruction, confirming that the wire had crossed the stenosis into the true lumen rather than creating a false passage. The absence of resistance during endoscope advancement and visualization of the esophageal lumen beyond the obstruction provided clinical confirmation of correct placement. Given the narrow working channel of the ultrathin endoscope (2.2 mm), standard dilation devices could not be deployed through the scope. The decision was therefore made to utilize the ultrathin endoscope itself as the dilating instrument. Dilation was carried out incrementally, given the friable nature of the irradiated tissue, and to minimize the risk of perforation during the initial session, with plans for further dilation at follow-up. The esophageal narrowing was ultimately dilated to 10 mm, with successful passage into the middle third of the esophagus (Figure 3).

**Figure 4:**
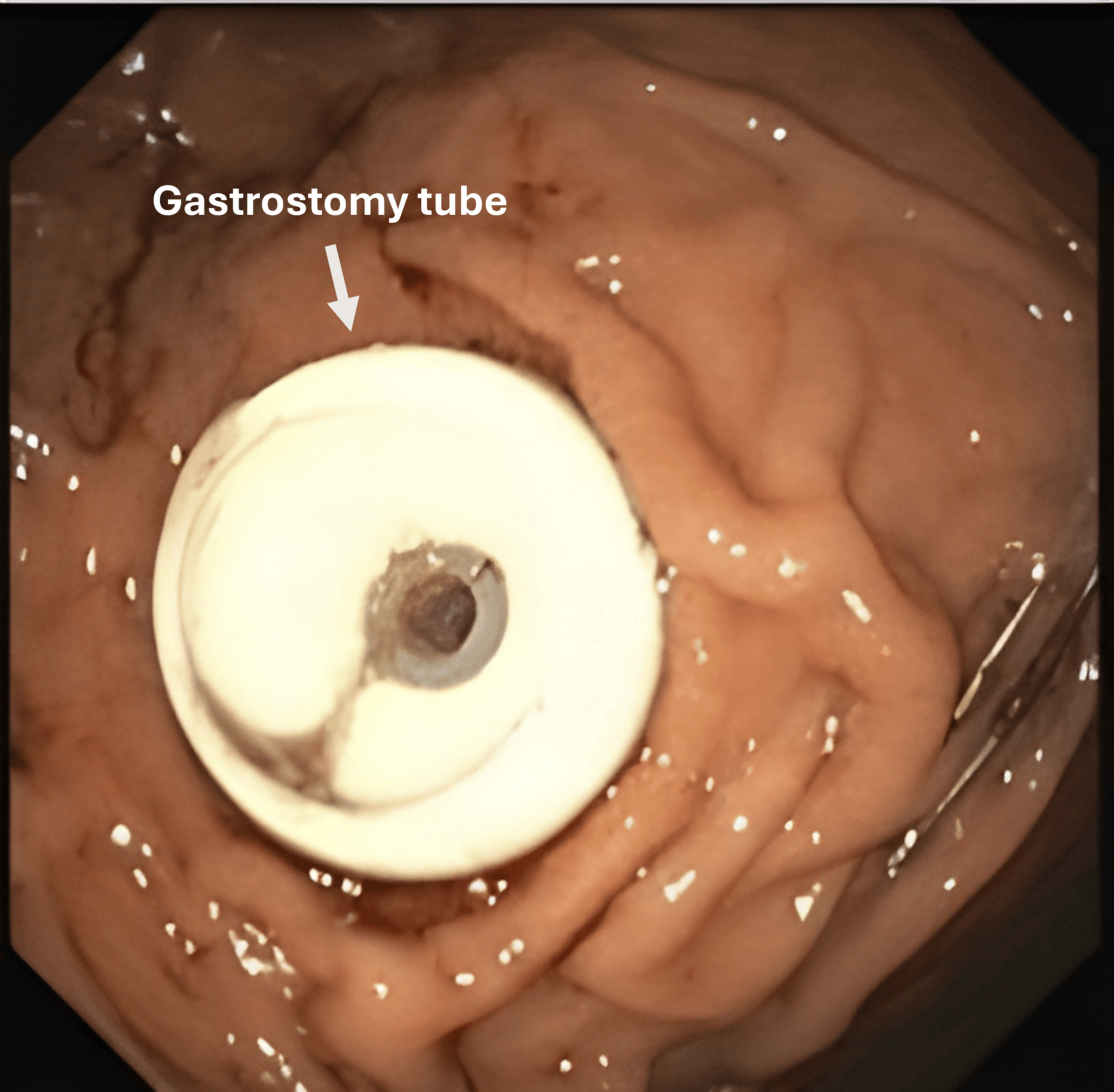
Successful PEG Tube Placement Intragastric view of the gastrostomy tube after successful placement. The arrow indicates the gastrostomy tube.

It was challenging to introduce a snare (2.5 mm) through the ultrathin endoscope, given its 2.2 mm channel; therefore, SpyBite biopsy forceps (1.2 mm; Boston Scientific Corporation, Marlborough, MA, USA) were used as an alternative. The guidewire was passed through the trocar and retrieved by the SpyBite forceps. The endoscope and forceps were removed, allowing the wire to be withdrawn orally. An EndoVive Safety gastrostomy tube (24 French, Boston Scientific) was lubricated, tied to the guidewire string, and advanced through the mouth and into the stomach (Figure 4).

The gastrostomy tube was then pulled out from the stomach through the skin. Correct positioning of the tube was confirmed by relook endoscopy. At his post-procedure follow-up, the patient tolerated his secretions better and was able to maintain a full liquid diet, with improvement in his cough. His weight increased from 134 to 147 pounds in one month. He was then scheduled for a repeat EGD in four weeks, with plans for further dilation to 14 mm and a trial to advance his diet, with the goal of making him PEG-independent.

## Discussion

Intraluminal esophageal obstruction can be caused by benign or malignant conditions. The most common benign cause is peptic strictures from long-standing gastroesophageal reflux disease [10]. Other common benign causes include corrosive substance ingestion, eosinophilic esophagitis, drug-induced esophagitis, or changes resulting from radiation or chemotherapy treatment [11]. For benign obstruction, the standard of care is endoscopic dilation using bougie or balloon techniques [5]. Malignant obstruction is typically managed with the endoscopic placement of SEMS [5]. For CEO, the preferred technique is CARD [12].

**Figure 5:**
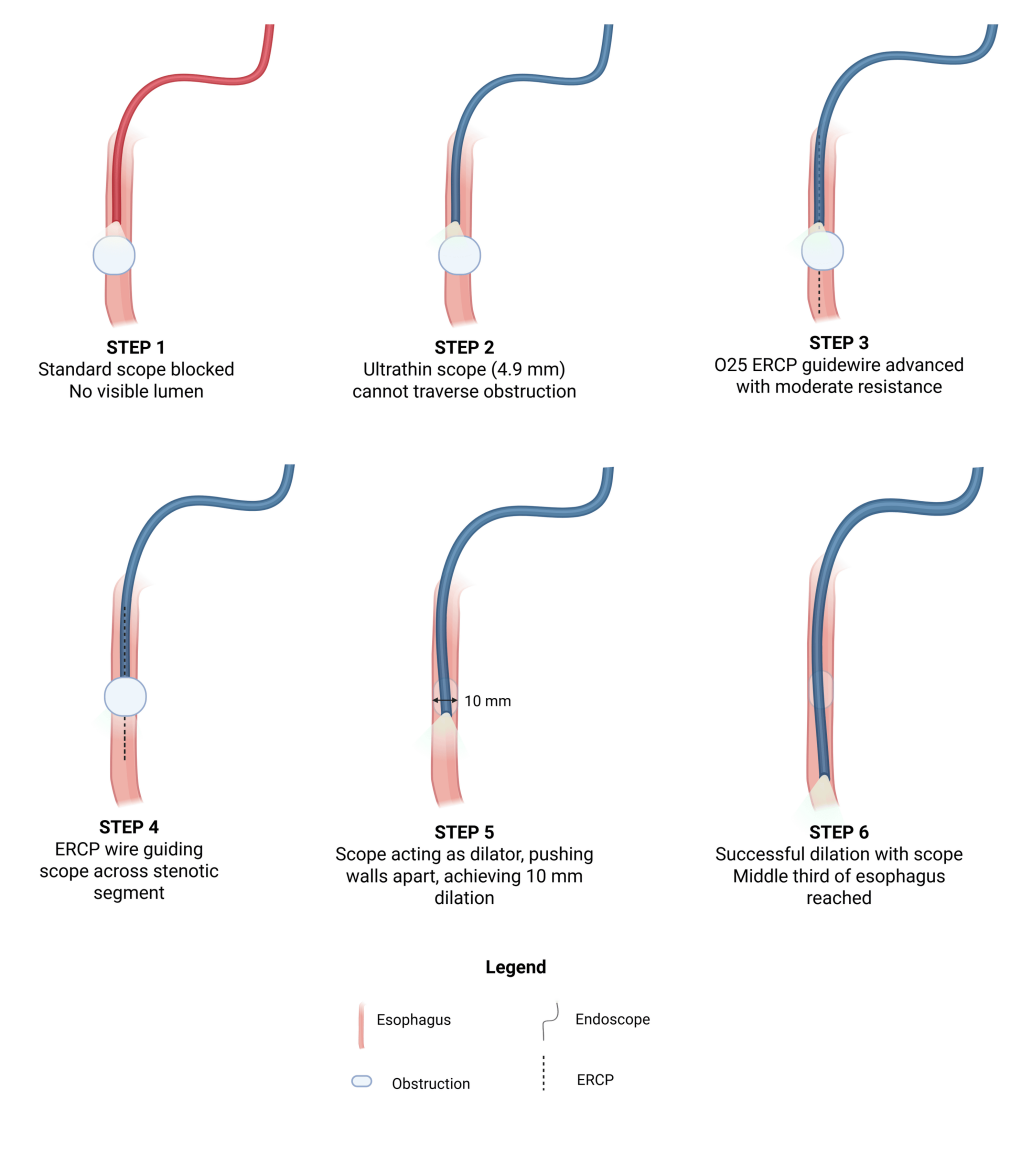
Stepwise Technique of Ultrathin Endoscope-Assisted Wire-Guided Dilation Schematic illustration of the six-step technique. Steps 1 and 2 demonstrate failed passage with standard and ultrathin endoscopes due to near-complete obstruction. Step 3 shows advancement of the O25 ERCP guidewire with moderate resistance. Step 4 demonstrates the guidewire guiding the ultrathin endoscope across the stenotic segment. Step 5 (key step) shows the ultrathin endoscope acting as the primary dilating instrument, pushing walls apart and achieving 10 mm dilation. Step 6 demonstrates successful passage into the middle third of the esophagus with restoration of esophageal patency. Created with BioRender.com, without the use of generative AI.

The use of ultrathin endoscopes in the management of esophageal strictures has been described in the literature, primarily as a tool for achieving access across tight strictures that cannot be traversed with a standard endoscope. Mulcahy et al. were among the first to describe this approach, reporting the successful passage of a 6 mm ultrathin endoscope through 9 of 12 esophageal strictures that were impassable with standard endoscopy, enabling subsequent dilation or nasogastric tube placement [13]. Kim et al. similarly described the use of ultrathin endoscopy to achieve correct guidewire placement across upper gastrointestinal obstructions, followed by separate balloon dilation or stent insertion [8]. More recently, Ekawati et al. reported a case series of 11 patients with corrosive injury-related esophageal strictures in whom ultrathin endoscopy was used to facilitate guidewire traversal, with subsequent dilation using TTS balloon or Savary bougie dilators [9]. Some cases in the literature also describe antegrade-only endoscopic recanalization performed by a single endoscopist without the retrograde component of CARD [14,15]; however, these procedures were typically performed under fluoroscopic guidance or with Savary and/or balloon dilators. Across all these reports, the ultrathin endoscope consistently served as an access tool rather than a therapeutic instrument, with separate dilation devices required to achieve luminal patency. In contrast, our case represents a distinct approach in which the ultrathin endoscope itself functioned as the primary dilating instrument, eliminating the need for separate dilation devices and further simplifying the procedure. To our knowledge, this is the first reported case of ultrathin endoscope-guided dilation for radiation-induced near-complete esophageal obstruction in which the endoscope itself served as the dilator (Figure 5).

We present a case of near-complete intraluminal esophageal obstruction due to post-radiation changes in the setting of hypopharyngeal cancer, successfully managed using an ERCP guidewire and an ultrathin endoscope as the dilating instrument. This approach allowed the patient to resume a liquid diet, improving his quality of life and nutritional status. The procedure was completed in 15 minutes without fluoroscopy, stenting, or advanced endoscopic equipment, suggesting potential advantages in procedure time and resource utilization compared to CARD, though direct comparisons cannot be drawn from a single case. Guidewire-dependent techniques such as ours may be limited in mature fibrostenotic strictures, where persistent myofibroblast activation and collagen cross-linking produce a rigid, non-compliant scar that is difficult to traverse. In contrast, earlier strictures, such as those seen in our patient, occurring within the first few months following acute radiation injury, often reflect ongoing inflammation and may be more amenable to guidewire traversal and endoscope-assisted dilation. Radiation-induced fibrosis typically develops 4-12 months after completion of therapy [16,17], and while the exact timing varies among patients, these intervals may serve as practical guidance for endoscopists in selecting the most appropriate intervention.

Our case illustrates the successful management of a challenging near-complete esophageal obstruction without the use of CARD or advanced interventions, including fluoroscopy, transillumination, balloon dilation, or stenting. While CARD is a safe and effective approach for CEO, retrospective data in head and neck cancer patients treated with radiation have documented complication rates as high as 36% per patient, though this estimate is derived from a retrospective study of 14 patients in which all complications occurred in those with complete rather than near-complete stenosis [18]. Nonetheless, they underscore the importance of identifying alternative approaches for this population. Patients with esophageal obstruction have a poor quality of life, as they rely on PEG tube feeds, which are associated with an increased risk of complications. In our case, the procedure was completed in 15 minutes without advanced equipment, and the patient subsequently tolerated a full liquid diet with significant weight gain, demonstrating meaningful short-term clinical benefit. While these findings are promising, conclusions regarding the safety, efficacy, and cost-effectiveness of this technique cannot be generalized from a single case. A formal barium esophagram was not performed prior to the procedure, and procedural documentation did not include the stricture length or distance from the incisors; these details would have better characterized the obstruction and are recommended for future reports of this technique. Further investigation is needed to optimize the technique and elucidate its long-term benefits, risks, limitations, and appropriate patient selection criteria.

## Conclusions

Near-complete esophageal obstruction following radiation therapy for head and neck cancers presents a significant clinical challenge, often requiring complex, resource-intensive interventions such as CARD. We present a case of successful management using an ERCP guidewire and an ultrathin endoscope as the dilating instrument, without the need for fluoroscopy, transillumination, stenting, or advanced endoscopic equipment. Unlike prior reports in which the ultrathin endoscope served as an access tool followed by separate mechanical dilation, in this case, the ultrathin endoscope itself functioned as the dilator, further simplifying the procedure. This technique may be most applicable to early inflammatory strictures rather than mature fibrostenotic ones, and findings are preliminary and hypothesis-generating. Further prospective studies are needed to validate this approach and establish long-term outcomes, but this case suggests that ultrathin endoscope-guided dilation may represent a feasible, practical alternative in appropriately selected patients, particularly in resource-limited settings where advanced interventional equipment may not be available.
